# Multiple genotypes of Echovirus 11 circulated in mainland China between 1994 and 2017

**DOI:** 10.1038/s41598-019-46870-w

**Published:** 2019-07-22

**Authors:** Jie Li, Dongmei Yan, Li Chen, Yong Zhang, Yang Song, Shuangli Zhu, Tianjiao Ji, Weimin Zhou, Fangrong Gan, Xianjun Wang, Mei Hong, Luyuan Guan, Yong Shi, Guizhen Wu, Wenbo Xu

**Affiliations:** 1 WHO WPRO Regional Reference Poliomyelitis Laboratory, National Institute for Viral Disease Control and Prevention, Chinese Center for Disease Control and Prevention, Beijing, China; 2 NHC Key Laboratory of Biosafety, National Institute for Viral Disease Control and Prevention, Chinese Center for Disease Control and Prevention, Beijing, China; 3 NHC Key Laboratory of Medical Virology, National Institute for Viral Disease Control and Prevention, Chinese Center for Disease Control and Prevention, Beijing, China; 4 Beijing Red Cross Blood Center, Beijing, China; 5 Shandong Center for Disease Control and Prevention, Jinan city, Shandong Province People’s Republic of China; 6 Tibet Center for Disease Control and Prevention, Lhasa city, Tibet Autonomous Region People’s Republic of China; 7 Shananxi Center for Disease Control and Prevention, Xi’an, Shananxi Province People’s Republic of China; 8 Jiangxi Center for Disease Control and Prevention, Nanchang, Jiangxi Province People’s Republic of China

**Keywords:** Genotype, Infection

## Abstract

Echovirus 11 (E-11) is one of the most frequently isolated enteroviruses causing meningitis and other diseases such as hand, foot, and mouth disease (HFMD) and acute flaccid paralysis (AFP). Fifty-nine newly determined E-11 *VP1* sequences from the China AFP and HFMD surveillance network and 500 E-11 *VP1* sequences obtained from the GenBank database, which were associated with 12 categories of diseases, were screened for phylogenetic analysis. Based on the standard method of genotype classification, E-11 strains circulated worldwide were reclassified into six genotypes as A, B, C, D, E, and F, in which genotype F is newly divided, and genotypes A and C are further divided into A1–5 and C1–4 by this research, whereas genotype D was still divided into D1–5 as in a previous study of Oberste *et al*. Sub-genotype A1 was the predominant sub-genotype in mainland China between 2008–2017, whereas sub-genotype D5 was the predominant sub-genotype circulated outside China from 1998–2014. However, genotype and sub-genotype spectra showed statistical significance among AFP and HFMD cases (χ^2^ = 60.86, P < 0.001), suggesting that different genotypes might have a tendency to cause different diseases. Strengthening the surveillance of E-11 might provide further information about pathogenic evolution or specific nucleotide mutation associated with different clinical diseases.

## Introduction

Human enteroviruses are small, nonenveloped, positive single-stranded RNA viruses that belong to genus *Enterovirus*, family *Picornaviridae*. Initially, the genus *Enterovirus*, capable of infecting humans, was divided into 66 serotypes by a neutralization assay that was used as the previous gold standard for enterovirus serotype identification. However, this method had some disadvantages, and no new enterovirus serotypes were confirmed for more than 20 years after the 1970s^1^. With the development of molecular typing methods, Oberste *et al*. first proposed a method for classification of enteroviruses based on molecular biological characterization that sub-grouped the enteroviruses into four species, EV-A to EV-D^2,3^. As molecular methods have become the current gold standard for enterovirus typing, more than 110 EV types have been identified^4,5^.

Echovirus was first isolated as a new type virus different from the poliovirus and coxsackievirus using tissue culture techniques in the early 1950s^6^. Because it can cause only a cytopathic effect with no pathogenicity to experimental animals, it is called an “enteric cytopathogenic human orphan virus”. In addition, echovirus, coxsackievirus group B, coxsackie A9 and several novel enteroviruses make up the EV-B species, which is the largest group of the *Enterovirus* genus, with 28 serotypes (http://www.picornaviridae.com/enterovirus/ev-b/ev-b.htm). Like other enteroviruses, E-11 infections are associated with a wide spectrum of illnesses, ranging from mild nonspecific symptoms to systemic disorders such as rash, febrile illness, HFMD, and uveitis to severe neurological disorders, including meningitis, encephalitis and AFP^7–9^. In particular, E-11 was reported to cause severe illnesses in neonates or infants, with high morbidity and mortality, causing great social panic^10–14^. In addition, E-11 can be transmitted vertically from mother to child, increasing the difficulty of controlling infections^15,16^. Furthermore, E-11 has frequently been identified as the causal agent of outbreaks, and reports about it could be found in countries such as India, Thailand, Japan, and Israel^17–19^.

As molecular typing methods based on entire *VP1* sequences have been broadly used and accepted, an increasing number of researchers have proposed different genotyping and sub-genotyping of E-11. The first molecular epidemiology study of E-11 based on entire *VP1* sequences showed that at least four monophyletic genotypes circulated among 16 countries worldwide from 1953–2001^20^. However, several studies tended to divide E-11 into four more genotypes and had some disagreements with the previous study regarding the continuous enrichment of E-11 genetic evolution^14,21,22^. In China, few studies on the molecular epidemiology of E-11 have been performed, and sequences used in the studies are not representative enough. Therefore, studies on the molecular epidemiology of E-11 in mainland China are indispensable and significant.

In this study, a total of 559 entire *VP1* sequences, including 500 (359 from abroad) sequences downloaded from GenBank and 59 sequences obtained from the China surveillance network in 11 provinces during the period from 1999 to 2017, were used as a dataset for molecular epidemiology. In this dataset, 94 sequences from both domestic regions and overseas were selected as representative sequences for phylogenetic analysis, and 200 entire indigenous *VP1* sequences isolated between 1994–2017 were used to describe the molecular epidemiology of E-11 in mainland China. The results of this study will provide important basic information about the genetic evolution of E-11 circulated in China as well as a deep understanding of its genetic characteristics and clinical pathogenicity.

## Results

### Geographic and temporal distribution of E-11 strains isolated in this study

A total of 59 strains isolated in this study are summarised in Supplementary Materials (Supplementary Table S1), among them, 18 were identified as E-11 found by the HFMD surveillance network from 2010 to 2013 and from 2015 to 2017. They were collected from mild HFMD cases except for four collected from severe HFMD cases in Hunan (1 strain in 2010), Hainan (1 strain in 2010), Guangdong (1 strain in 2012) and Hebei provinces (1 strain in 2017). The other 41 strains were identified from the AFP case surveillance in Shandong Province (26 isolates) between 1999 and 2003 and that in the Tibet Autonomous Region (15 isolates) in 1999. The entire *VP1* regions of these 59 isolates in this study shared 76.5–80.4% nucleotide sequence similarity and 88.0–94.1% amino acid similarity respectively with the prototype strain Gregory, and 85.2–94.5% nucleotide sequence similarity and 72.6–80.5% amino acid similarity respectively with the prime strain Silva.

### Six E-11 genotypes were assigned based on entire *VP1* sequences

**Figure 1:**
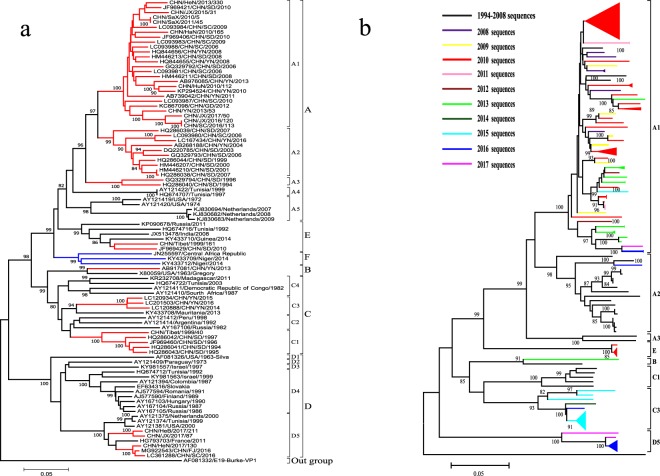
(**a**) Phylogenetic analysis. Phylogenetic dendrogram generated by the neighbour-joining method using the maximum composite likelihood model based on complete E-11 *VP1* nucleotide sequences of 94 representative strains from both domestic and overseas sources between 1953 (the prototype strain Gregory) and 2017, with E-19 strain Burke as the outgroup. A difference of at least 8% and 15% in the entire *VP1* region of E-11 strains was used to distinguish sub-genotypes and genotypes. The numbers at nodes represent the percentage of 1000 bootstrap replicates that supported the distal cluster. The name formatting of E-11 strains followed “GenBank number/country of origin/year of isolation”. Genotypes and sub-genotypes were marked on the right side of the tree. The relative phylogenetic distance was measured by the scale at the bottom, which means Nucleotide substitution rate, the 0.05 indicates that there are 5 differences per 100 nucleotides when the length of the branch is equal to the scale at the bottom. Representative strains isolated in China are marked in red, and the newly added genotype F is marked in blue. (**b**) Phylogenetic tree based on the complete *VP1* nucleotide sequences of 200 E-11 strains in mainland China. The branches of sequences are highlighted in different colours according to the year (2008 to 2017).

**Figure 2:**
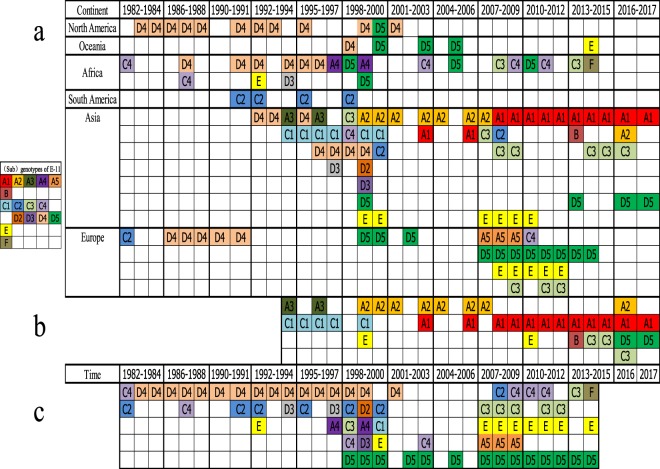
Time distribution of different (sub-)genotypes from 1982–2017. Note: (**a**) shows the (sub-)genotype distribution of different continents based on 559 E-11 sequences. (**b**) Shows the time distribution of different (sub-)genotypes of E-11 based on 200 sequences isolated in mainland China. (**c**) Shows the time distribution of different E-11 (sub-)genotypes based on 359 sequences isolated outside mainland China.

In total, 500 entire E-11 *VP1* sequences were retrieved from GenBank (sequences deposited before December 5th, 2018). These *VP1* sequences were isolated from 34 countries of six continents from 1953 to 2016, including the prototype strain Gregory. In addition to 59 strains isolated in this study, a dataset of 559 entire E-11 *VP1* sequences was formed to screen for representative sequences. The representative sequences were selected as per the following rules: covering most of the countries and time ranges and not having sequences with high similarity or with significant errors. In all, 94 sequences were selected to generate a phylogenetic tree (Fig. 1a) and analyse the (sub-)genotype distribution (Fig. 2a).

Based on the phylogenetic dendrogram (Fig. 1a) and following a previous study, all the E-11 strains could be segregated into 6 distinct genotypes, A, B, C, D, E, and F, sharing at least 85% *VP1* nucleotide sequence identity (Table 1). Genotype F was considered to be a new genotype generated from the evolution of E-11 strains that were isolated in landlocked countries in central and western Africa. With the continuous enrichment of E-11 isolates, genotypes A, C and D could be further subdivided into A1–5, C1–4 (by this research), and D1–5 (by previous research) sub-genotypes with a difference of more than 8% in the entire *VP1* region. The prototype strain Gregory, which was isolated in the USA from a healthy person in 1953, clustered with a Chinese strain isolated from a healthy child in Yunnan province in 2013 to form genotype B (using the genotype name from its first study), which differed from other genotypes by 19.5–24.3% (Table 1) and was more similar (19.8%) to genotype C than to the others. Notably, sub-genotypes A1, A2 and A3 were isolated in only mainland China, whereas three strains isolated from HFMD cases in China in 2017 belonged to the D5 sub-genotype and were most similar to a strain isolated from a cerebrospinal fluid sample of a patient with meningitis in France in 2011. This was the first report of sub-genotype D5 of E-11 isolated from HFMD cases in mainland China.

**Table 1:** The average divergence between different genotypes.

Genotype	A	B	C	D	E	F
A	**0.128** ± **0.006**	0.091	0.081	0.127	0.054	0.059
B	*0.224*	**0.123** ± **0.011**	0.073	0.129	0.091	0.089
C	*0.219*	*0.198*	**0.165** ± **0.008**	0.116	0.08	0.082
D	*0.249*	*0.242*	*0.239*	**0.125** ± **0.007**	0.111	0.106
E	*0.193*	*0.220*	*0.219*	*0.237*	**0.122** ± **0.007**	0.032
F	*0.202*	*0.228*	*0.225*	*0.243*	*0.188*	**0.159** ± **0.010**

Note: The bottom left data in italics indicate nucleotide diversity. The top right data in normal font indicate deduced amino acid sequence diversity. Mean nucleotide diversities within every genotype are marked in bold and underlined.

### Multiple genotypes of E-11 circulated in mainland China

A total of 200 sequences were determined from four different sources (people with AFP, people with HFMD, healthy children and sewage) in 12 provinces of mainland China over the time period from 1994 to 2017, and the largest number of E-11 sequences was identified from AFP-related samples. These strains belonged to sub-genotypes A1–3, C1, C3, and D5 and genotypes B and E. The isolation sources and time distribution of the genotype are shown in Table 2. The sub-genotype A1 was isolated first from AFP cases in 2003 and has been persistently circulated in nine provinces of mainland China from 2008 to 2017. However, the emerging sub-genotype D5 was detected in HFMD cases and healthy children in 2016–2017 in five provinces, which is the first report of sub-genotype D5 being isolated from HFMD cases in mainland China.

**Table 2:** Summary of 200 E-11 strains of mainland China.

Source of specimen	(Sub-)genotype (number of isolates)	Isolation year	Province
AFP	A1(20)	2003, 2006, 2008–2010	Shandong, Sichuan
	A2(37)	1999–2001, 2003–2004, 2006–2007	Shandong, Yunnan, Sichuan
	A3(2)	1994, 1996	Shandong
	C1(15)	1994–1995, 1997, 1999–2000	Shandong, Tibet
	E (4)	1999	Tibet
HFMD	A1(16)	2010–2013, 2015–2017	Yunnan, Hunan, Shananxi, Hainan, Guangdong, Henan, Jiangxi, Sichuan
	D5(5)	2016–2017	Fujian, Jiangxi, Henan, Hebei
Sewage	A1(55)	2010–2013	Shandong, Yunnan
	C1(1)	1996	Shandong
	E (8)	2010	Shandong
Healthy children	A1(8)	2008, 2011, 2013, 2016	Yunnan
	A2(1)	2016	Yunnan
	B (1)	2013	Yunnan
	C3(19)	2014–2016	Yunnan
	D5(8)	2016	Sichuan

A phylogenetic tree was also constructed using the same method and 200 entire *VP1* sequences isolated from mainland China to analyse molecular epidemiological characteristics (Fig. 1b), wherein the times of the strains isolation were marked in different colours; we found the sub-genotype A1 had a wide time distribution, whereas sub-genotypes A3 and C1 disappeared before 2008. Genotype E was not found after 2010, but sub-genotypes C3 and D5 became the most frequently detected sub-genotypes in four recent years. Moreover, we learned that the sub-genotype A2 was mostly isolated from AFP cases before 2008 but was recently isolated from a healthy child in Yunnan province in 2016. Additional detailed information about the time and (sub-) genotype circulation in mainland China is shown in Fig. 2b, and great shifts in persistent circulating (sub-)genotypes were found in 1999 (sub-genotype C1 to A2) and 2008 (sub-genotype A2 to A1).

After the above analysis, we could conclude that the sub-genotypes A1 and D5 might have a wide geographical distribution, suggesting a strong transmission ability. Time analysis showed that sub-genotypes A1, C3, and D5 had been circulated in mainland China in four recent years. Furthermore, sub-genotype A1 had wide time and geographical distributions and was the absolute dominant sub-genotype in mainland China during the time period from 2008–2017.

### Genotypes and sub-genotypes of E-11 that circulated outside mainland China

A total of 359 out 500 strains retrieved from GenBank were isolated from 33 countries of six continents in the time period of 1953 to 2014, and they were associated with 11 categories of diseases (Fig. 2c and Supplementary Table S2). After genotyping, those strains were distributed in B and F genotypes and A4–5, C2, C4, and D1–5 sub-genotypes. From Fig. 2c and Supplementary Table S2, we obtained the graphical genotype distribution of E-11 for each continent and its time-dependent circulation pattern: geographically, the sub-genotype A5 has been reported in only North America and Europe, and the sub-genotype D5 has been detected in all continents except South America. The new genotype F, and the sub-genotype A4 were found in only Africa. However, the continent of South America had only sub-genotype C2 been reported. Furthermore, the sub-genotype D5 had first been isolated in Tunisia in 1998 and had a persistent epidemic period lasting until 2014; D5 was the predominant sub-genotype outside of China from 1998 to 2014. From Fig. 2c, the circulation pattern of E-11 outside of China greatly shifted in 1998, the persistent circulating sub-genotype switched from D4 to D5.

### Analysis of nucleotide and amino acid variation of entire *VP1* region in the sub-genotype A1 of E-11 isolated in mainland China and associated with AFP and HFMD

Among 200 strains of E-11 that circulated in mainland China, 99 strains were isolated and detected in the AFP and HFMD surveillance network (Table 2). Strains of E-11 isolated from AFP cases were genotypes A and E and sub-genotype C1, whereas strains isolated from HFMD cases were sub-genotypes A1 and D5. The distribution of different genotypes or sub-genotypes of E-11 had statistical significance among AFP and HFMD cases (χ^2^ = 60.86, P < 0.001). To explore the reasons for sub-genotype A1 causing different disease manifestations, we compared the nucleotide and amino acid mutations of the entire *VP1* region of sub-genotype A1 between AFP and HFMD cases (Table 3). The results showed that nucleotide positions of 161, 439 and 831 had mutations resulting in transitions from histidine (H) to arginine (R), isoleucine (I) to valine (V) and serine (S) to asparagine (N) at amino acid positions 54, 147 and 277, respectively.

**Table 3:** The statistical analysis and nucleotide, amino acid mutations in the entire *VP1* region of sub-genotype A1 among HFMD and AFP cases.

Disease type	Total number of sequences of different sub-genotype		Nucleotide	Amino acid	Nucleotide	Amino acid	Nucleotide	Amino acid
	A1	D5	161/876^*^	54/292^*^	439/876^*^	147/292^*^	831/876^*^	277/292^*^
HFMD	16	5	A → G	H → R	A → G	I → V	C → T	S → N
AFP	20	0	0 (0%)	0 (0%)	5 (31.25%)	5 (31.25%)	8 (50%)	8 (50%)
χ^2^(*P*)	60.86 (*P* < *0.001*)	6 (30%)	6 (30%)	0 (0%)	0 (0%)	1 (5%)	1 (5%)	

The A/B^*^ format indicates nucleotide or amino acid variation site/full length of nucleotide or amino acid sequence.

## Discussion

The E-11 virus, within species EV-B, is one of the most commonly isolated human enteroviruses. Reports of its separation rate originate from both China and abroad^23–25^, and data from the AFP surveillance system revealed that EV-B and especially echoviruses may be the most frequently detected non-polio enteroviruses (NPEVs) in AFP cases of mainland China^26,27^. Through retrospective research, we found that clinical symptoms caused by E-11 covered a wide range of feature; however, the dynamic prevalence and extinction patterns of E-11 domestic and abroad were not clear. Therefore, combining the E-11 sequences available worldwide to obtain global epidemic trends is inevitable and indispensable, and the Chinese surveillance system for AFP and HFMD would make a very large contribution to this research.

At present, the unified identification of E-11 genotypes and sub-genotypes is still uncertain and unclear. Initial research by Oberste *et al*. had divided E-11 strains into four independent genotypes, and because of the limited number of isolates, only genotype D was further subdivided^20^. However, domestic research on E-11 is mostly limited to a single surveillance system in one province or region and is based on only partial *VP1* sequences when phylogenetic trees are constructed for genotyping and sub-genotyping. In addition, studies have suggested that strains prevalence may be associated with climate factors such as high temperature and high humidity^28,29^. As China is a vast country, different regions may have different terrains and climates, and the E-11 strains circulated in each region may have some indigenous characteristics. Therefore, a comprehensive study of E-11 molecular epidemiology in mainland China with a larger geographical scale and wider time span than those in this study is indispensable and important.

The E-11 sequences obtained in this study were from the entire *VP1* region of 876 nucleotides, and the sequences downloaded from GenBank were carefully screened, finally resulting in 94 representative strains that were selected as representative of genotypes and sub-genotypes based on the “gold standard” recognized internationally and the rationale for this has been summarized in previous research^20–22,30^. The tree diagram for genotyping showed that Chinese isolates and foreign isolates converged in different branches, suggesting a difference between Chinese isolates and foreign isolates. In addition, the pattern of worldwide E-11 circulation was different from the corresponding pattern in mainland China. D5 was the predominant sub-genotype circulated outside of China from 1998 to 2014, whereas A1 was the predominant sub-genotype in mainland China between 2008 and 2017. Moreover, a comprehensive molecular epidemiology study of E-11 was conducted in 12 provinces in mainland China with a long-term timescale over the period from 1994 to 2017; the resulting data is of great significance as they help us understand the time, geographical distribution, and pathogenicity of E-11 circulated in mainland China.

The differences between the entire *VP1* nucleotide and amino acid sequences of all Chinese strains in our experiment and those of the prototype strain Gregory are 19.6–23.5% and 5.9–12.0%, respectively, the large differences are consistent with the results from previous studies^20,31,32^. Moreover, we also compared the strains isolated in our study with the prime strain Silva and found that the differences in nucleotide (19.5–27.4%) and amino acid sequences (5.5–14.8%) are similar to the differences between the sequences of the strains in this study and Gregory sequences, revealing that the sequences from this study may belong to an intermediate phenotype.

The phylogenetic analysis indicated that the E-11 strains could be divided into six genotypes, described as A, B, C, D, E and F, and that genotype F was a new genotype produced during the evolution of E-11. In addition, genotypes A, C and D could be further subdivided into A1–5, C1–3 and D1–5. Genotype D was divided based on the study of Oberste *et al*., and because genotype A is the absolute dominant genotype in China, the division of its sub-genotypes is basically based on domestic research^32–34^. The five genotypes other than genotype F had representative strains isolated in mainland China; the E-11 strains isolated from the HFMD surveillance system in 2016–2017 were particularly classified into genotype D and sub-genotype D5, which was most similar to a strain isolated from a cerebrospinal fluid sample of a patient in France in 2011 with meningitis^35^. In the same year, there was a report on clinical cases of meningitis caused by sub-genotype D5 in Fujian Province, China^36^. However, the origin of the sub-genotype D5 in mainland China and the reason for its detection in HFMD cases many years later, in the year 2016, remain unknown. Although the sub-genotype D5 has been isolated in only two recent years (2016–2017), the geographic extent of its dissemination was extensive, which might be related to genotype D showing wide geographical distribution. This phenomenon warns us that further pathogen monitoring must be strengthened to prevent the genotype D strain from spreading extensively in mainland China.

Analysis of 200 strains isolated from mainland China showed that the genotypes and sub-genotypes circulated in mainland China during 1994–2017. The strain isolated from the faeces of a healthy child in Yunnan province of China in 2013, together with the prototype strain Gregory, constitutes genotype B, and the difference between these two strains is 12.3% less than the differences within other genotypes. This “return to the ancestors” phenomenon might be caused by coincidental virus mutation. Moreover, statistical data indicated that different genotypes might have a tendency to cause different diseases and that genotypes A and E and sub-genotypes C1 and D5 might be pathogenic genotypes causing AFP or HFMD. Furthermore, analysis of mutations in the complete *VP1* region of sub-genotype A1 among AFP and HFMD cases revealed three significant nucleotide mutations causing three corresponding amino acid mutations. As the *VP1* region plays an indispensable role in mediating receptor binding and changes in the receptor might directly affect tissue tropism to produce different clinical symptoms^37,38^, these mutations might change some structure or function of the VP1 protein.

Time analysis showed that sub-genotypes A1–3, C1, C3, and D5 and genotypes B and E had been circulated in mainland China from 1994 to 2017. With evolution over time, a small amount of sub-genotypes A3 and C1 transiently appeared, and the emerging sub-genotypes C3 and D5 began to circulate in mainland China, suggesting that the pattern of E-11 circulation in mainland China had changed, but the reason remained unknown. Research on the geographical distribution of each genotype showed that the sub-genotypes A1 and D5 had a wide geographical distribution, whereas temporal evidence and geographical distribution analysis showed that the sub-genotype A1 was absolutely the dominant genotype in mainland China. However, determining whether the emerging sub-genotype D5 existed in mainland China for a long time requires continuous surveillance.

Based on the current research, we have a preliminary understanding of the genotyping and sub-genotyping of E-11 on the global scale and have obtained basic information on the molecular epidemiological characteristics of E-11 circulated in mainland China. However, the disease burden of E-11 worldwide was underestimated because of the incompleteness of a surveillance system. Therefore, this study has crucial public significance and practical utility for disease control and prevention. We will continue to conduct whole-genome sequencing analysis to obtain more information about the variability and genetic recombination of E-11 in mainland China.

## Materials and Methods

### Sample collection and virus isolation

The 59 E-11 strains used in this study were isolated from patients with HFMD (18 isolates) or AFP (41 isolates) in the 11 provinces Hebei, Shandong, Guangdong, Hainan, Shaanxi, Sichuan, Yunnan, Henan, Hunan, Jiangxi and Tibet between 1999 and 2017. All of those clinical samples (stool, throat swabs, and nasal swabs) were collected according to the national HFMD guidelines (http://www.gov.cn/gzdt/2009-06/04/content_1332078.htm) and the national AFP surveillance guidelines (http://www.moh.gov.cn/zwgk/jdjd/201304/3825417a79574da0b7ca44bfeef2b76b.shtml). A commercial real-time PCR assay (Shuoshi Biotech, Jiangsu, China) was used to screen for EV-A71, CV-A16, and other EVs as in a previous study^39^. Cell lines for virus propagation and purification of the other EV-positive samples included human rhabdomyosarcoma (RD) and human laryngeal epidermoid carcinoma (HEp-2) cell lines, which were obtained from the WHO Global Poliovirus Specialized Laboratory, USA, and were originally purchased from the American Type Culture Collection. Infected cell cultures were harvested after complete cytopathic effect (CPE) was observed and maintained frozen (−40 °C) for long-term storage.

### Determination of the entire* VP1* nucleotide sequence of E-11

Viral RNA was extracted from 200 µl of viral-infected culture supernatant using a Tianlong RNA/DNA extraction kit (Tianlong Science & Technology, China). Reverse transcription polymerase chain reactions (RT-PCRs) were performed to amplify the entire *VP1* capsid region (876 nucleotides) using a PrimeScript One Step RT-PCR Kit Ver. 2 (TaKaRa, Dalian, China) with primers designed in this study (upstream primer E-11-2293-S: 5′-GCTGGTAATGTGACGTGCTG-3′ and downstream primer E-11-3416-A: 5′-TCGTCCCACACACAGTTTTG-3′) and the previously described primers 490 and 493^40^. The PCR conditions were as follows: 50 °C for 30 min; 94 °C for 3 min; 32 cycles at 94 °C for 30 s, 50 °C for 30 s and 72 °C for 1 min and 20 s; and a final extension step at 72 °C for 10 min. The PCR products were analysed by 1.5% agarose gel electrophoresis, and positive products were purified using the QIAquick PCR Purification Kit (Qiagen, Germany); both strands of the amplicons were then sequenced using the ABI 3130 Genetic Analyzer (Applied Biosystems, USA)^41^.

### Phylogenetic and statistical analysis

The dataset used in this study include 500 entire E-11 *VP1* sequences (359 from abroad, 141 from China) obtained from GenBank before December 5th, 2018, and 59 entire E-11 *VP1* sequences (18 isolated from HFMD cases, 41 isolated from AFP cases) determined in this study. Among all these E-11 sequences, 94 representative E-11 strains (including 54 Chinese strains and 40 international strains) that were located in 18 countries from 1953 to 2017 were selected for genotyping and sub-genotyping based on previous studies and the topological structure of their dendrograms. In addition, 200 entire *VP1* sequences (141 of GenBank) from mainland China were used to describe the molecular epidemiology of E-11 in mainland China.

The ClustalW tool in MEGA 7.0.26 was used for sequence alignment, and a bootstrap test with 1,000 replications was used to test the robustness of the constructed phylogenies^42^. Phylogenetic dendrograms based on the entire *VP1* coding sequence were generated by neighbour-joining with the maximum composite likelihood model. Bootstrap values greater than 80% were considered statistically significant for grouping^43^. In addition, a difference of at least 15% between groups and 8% within groups in the *VP1* region, determined by computing the group mean distance in MEGA, was used to distinguish genotypes and sub-genotypes^44–47^. The similarity of E-11 sequences between the prototype strain (primer strain) and isolated strains in this study was determined by Bioedit software. Statistical analysis was performed using SAS 9.4. The difference in the distribution of genotypes or sub-genotypes between disease types was analysed using a chi-square test. P values less than 0.05 were considered statistically significant.

### Ethics statement

This study was not involved to human experimentation or human participants. The only human materials used were stool samples and throat swab samples collected from HFMD and AFP patients, which for public health purposes according to the national HFMD or AFP guidelines. Written informed consent for the use of their clinical sample was obtained from the parents of the children. This study was approved by the Ethics Review Committee of the National Institute for Viral Disease Control and Prevention (NIVDC), Chinese Center for Disease Control and Prevention. All experimental protocols were approved by the NIVDC, and the methods were carried out in accordance with the approved guidelines.

### Nucleotide sequence accession numbers

The 12 representative nucleotide sequences in this study have been deposited in GenBank under accession numbers MK359992-MK360003.

## Supplementary information

Supplementary Table S1

Supplementary Table S2

Supplementary legends
