# Supplementary material for: Multiple genotypes of Echovirus 11 circulated in mainland China between 1994 and 2017
Source: Sci Rep. 2019 Jul 22;9:10583. doi: 10.1038/s41598-019-46870-w (PMC6646367; doi:10.1038/s41598-019-46870-w)
Supplement: Supplementary file 1 — Supplementary Table S1 [file 41598_2019_46870_MOESM1_ESM.docx]

Multiple genotypes of Echovirus 11 circulated in mainland China between 1994 and 2017

Jie Li^1,2^, Dongmei Yan^1,2^, Li Chen^4^, Yong Zhang^1,2^, Yang Song^1^, Shuangli Zhu^1^, Tianjiao Ji^1^, Weimin Zhou^2^, Fangrong Gan^2^, Xianjun Wang^5^, Mei Hong^6^, Luyuan Guan^7^, Yong Shi^8^, Guizhen Wu^2^*, Wenbo Xu ^1,3^*

^1^WHO WPRO Regional Reference Poliomyelitis Laboratory, National Institute for Viral Disease Control and Prevention, Chinese Center for Disease Control and Prevention, Beijing, China.

^2^ NHC Key Laboratory of Biosafety, National Institute for Viral Disease Control and Prevention, Chinese Center for Disease Control and Prevention, Beijing, China.

^3^ NHC Key Laboratory of Medical Virology, National Institute for Viral Disease Control and Prevention, Chinese Center for Disease Control and Prevention, Beijing, China.

^4^Beijing Red Cross Blood Center, Beijing, China.

^5^Shandong Center for Disease Control and Prevention, Jinan city, Shandong Province, People’s Republic of China.

^6^Tibet Center for Disease Control and Prevention, Lhasa city, Tibet Autonomous Region, People’s Republic of China.

^7^Shananxi Center for Disease Control and Prevention, Xi’an, Shananxi Province, People’s Republic of China.

^8^Jiangxi Center for Disease Control and Prevention, Nanchang, Jiangxi Province, People’s Republic of China.

*Address correspondence to Guizhen Wu and Wenbo Xu

Guizhen Wu:

NHC Key Laboratory of Biosafety, National Institute for Viral Disease Control and Prevention, Chinese Center for Disease Control and Prevention, Beijing, China.

Mailing address: No. 155, Changbai Road, Changping District, Beijing 102206,

People’s Republic of China. Tel no. 0086-10-58900656, Fax no. 0086-10-58900657

Email: wgzcdc@hotmail.com

Wenbo Xu:

WHO WPRO Regional Reference Poliomyelitis Laboratory, NHC Key Laboratory of Medical Virology, National Institute for Viral Disease Control and Prevention, Chinese Center for Disease Control and Prevention, Beijing, China. Mailing address: No. 155, Changbai Road, Changping District, Beijing 102206, People’s Republic of China, Tel no. 0086-10-58900187

Fax no. 0086-10-58900187, Email: wenbo_xu1@aliyun.com

**Supplementary Table S1. Fifty-nine strains isolated in this study**

Note: severe cases were marked in bold.

**Supplementary Table S2. 359 E-11 isolates obtained outside of mainland China.**
