# Supplementary material for: Multiple genotypes of Echovirus 11 circulated in mainland China between 1994 and 2017
Source: Sci Rep. 2019 Jul 22;9:10583. doi: 10.1038/s41598-019-46870-w (PMC6646367; doi:10.1038/s41598-019-46870-w)
Supplement: Supplementary file 2 — Supplementary Table S2 [file 41598_2019_46870_MOESM2_ESM.docx]

Multiple genotypes of Echovirus 11 circulated in mainland China between 1994 and 2017

Jie Li^1,2^, Dongmei Yan^1,2^, Li Chen^4^, Yong Zhang^1,2^, Yang Song^1^, Shuangli Zhu^1^, Tianjiao Ji^1^, Weimin Zhou^2^, Fangrong Gan^2^, Xianjun Wang^5^, Mei Hong^6^, Luyuan Guan^7^, Yong Shi^8^, Guizhen Wu^2^*, Wenbo Xu ^1,3^*

^1^ WHO WPRO Regional Reference Poliomyelitis Laboratory, National Institute for Viral Disease Control and Prevention, Chinese Center for Disease Control and Prevention, Beijing, China.

^2^ NHC Key Laboratory of Biosafety, National Institute for Viral Disease Control and Prevention, Chinese Center for Disease Control and Prevention, Beijing, China.

^3^ NHC Key Laboratory of Medical Virology, National Institute for Viral Disease Control and Prevention, Chinese Center for Disease Control and Prevention, Beijing, China.

^4^Beijing Red Cross Blood Center, Beijing, China.

^5^Shandong Center for Disease Control and Prevention, Jinan city, Shandong Province, People’s Republic of China.

^6^Tibet Center for Disease Control and Prevention, Lhasa city, Tibet Autonomous Region, People’s Republic of China.

^7^Shananxi Center for Disease Control and Prevention, Xi’an, Shananxi Province, People’s Republic of China.

^8^Jiangxi Center for Disease Control and Prevention, Nanchang, Jiangxi Province, People’s Republic of China.

*Address correspondence to Guizhen Wu and Wenbo Xu

Guizhen Wu:

NHC Key Laboratory of Biosafety, National Institute for Viral Disease Control and Prevention, Chinese Center for Disease Control and Prevention, Beijing, China.

Mailing address: No. 155, Changbai Road, Changping District, Beijing 102206,

People’s Republic of China. Tel no. 0086-10-58900656, Fax no. 0086-10-58900657

Email: wgzcdc@hotmail.com

Wenbo Xu:

Regional Reference Poliomyelitis Laboratory, NHC Key Laboratory of Medical Virology, National Institute for Viral Disease Control and Prevention, Chinese Center for Disease Control and Prevention, Beijing, China. Mailing address: No. 155, Changbai Road, Changping District, Beijing 102206, People’s Republic of China, Tel no. 0086-10-58900187

Fax no. 0086-10-58900187, Email: wenbo_xu1@aliyun.com

**Supplementary Table S1. Fifty-nine strains isolated in this study**

| Isolated year | Provinces | Strains name of E-11 | case classification | Source of specimen |
| --- | --- | --- | --- | --- |
| 1999 | Tibet | CHN-Tibet-9909 | Not available | AFP |
| 1999 | Tibet | CHN-Tibet-99037 | Not available | AFP |
| 1999 | Tibet | CHN-Tibet-99038 | Not available | AFP |
| 1999 | Tibet | CHN-Tibet-99040 | Not available | AFP |
| 1999 | Tibet | CHN-Tibet-99044 | Not available | AFP |
| 1999 | Tibet | CHN-Tibet-99045 | Not available | AFP |
| 1999 | Tibet | CHN-Tibet-99046 | Not available | AFP |
| 1999 | Tibet | CHN-Tibet-99047 | Not available | AFP |
| 1999 | Tibet | CHN-Tibet-99050 | Not available | AFP |
| 1999 | Tibet | CHN-Tibet-99051 | Not available | AFP |
| 1999 | Tibet | CHN-Tibet-99122 | Not available | AFP |
| 1999 | Tibet | CHN-Tibet-99123 | Not available | AFP |
| 1999 | Tibet | CHN-Tibet-99143 | Not available | AFP |
| 1999 | Tibet | CHN-Tibet-99161 | Not available | AFP |
| 1999 | Tibet | CHN-Tibet-99163 | Not available | AFP |
| 1999 | Shandong | CHN-SD-99-1 | Not available | AFP |
| 1999 | Shandong | CHN-SD-99-2 | Not available | AFP |
| 2000 | Shandong | CHN-SD-00-1 | Not available | AFP |
| 2000 | Shandong | CHN-SD-00-2 | Not available | AFP |
| 2000 | Shandong | CHN-SD-00-3 | Not available | AFP |
| 2000 | Shandong | CHN-SD-00-4 | Not available | AFP |
| 2000 | Shandong | CHN-SD-00-5 | Not available | AFP |
| 2000 | Shandong | CHN-SD-00-6 | Not available | AFP |
| 2000 | Shandong | CHN-SD-00-7 | Not available | AFP |
| 2000 | Shandong | CHN-SD-00-8 | Not available | AFP |
| 2000 | Shandong | CHN-SD-00-9 | Not available | AFP |
| 2000 | Shandong | CHN-SD-00-10 | Not available | AFP |
| 2000 | Shandong | CHN-SD-00-11 | Not available | AFP |
| 2000 | Shandong | CHN-SD-00-12 | Not available | AFP |
| 2000 | Shandong | CHN-SD-00-13 | Not available | AFP |
| 2000 | Shandong | CHN-SD-00-14 | Not available | AFP |
| 2000 | Shandong | CHN-SD-00-15 | Not available | AFP |
| 2001 | Shandong | CHN-SD-01-1 | Not available | AFP |
| 2001 | Shandong | CHN-SD-01-2 | Not available | AFP |
| 2003 | Shandong | CHN-SD-03-3 | Not available | AFP |
| 2003 | Shandong | CHN-SD-03-5 | Not available | AFP |
| 2003 | Shandong | CHN-SD-03-7 | Not available | AFP |
| 2003 | Shandong | CHN-SD-03-8 | Not available | AFP |
| 2003 | Shandong | CHN-SD-03-9 | Not available | AFP |
| 2003 | Shandong | CHN-SD-03-10 | Not available | AFP |
| 2003 | Shandong | CHN-SD-03-11 | Not available | AFP |
| 2010 | Hunan | HuN-2010-112 | **severe case** | HFMD |
| 2010 | Shananxi | Sax-2010-5 | mild cases | HFMD |
| 2010 | Shananxi | Sax-2010-6 | mild cases | HFMD |
| 2010 | Hainan | HaN-2010-165 | **severe case** | HFMD |
| 2011 | Shananxi | Sax-2011-45 | mild cases | HFMD |
| 2011 | Shananxi | Sax-2011-47 | mild cases | HFMD |
| 2011 | Shananxi | Sax-2011-48 | mild cases | HFMD |
| 2012 | Guangdong | GD-2012-34 | **severe case** | HFMD |
| 2013 | Yunnan | YN-2013-53 | mild cases | HFMD |
| 2013 | Henan | HeN-2013-330 | mild cases | HFMD |
| 2015 | Jiangxi | JX-2015-31 | mild cases | HFMD |
| 2016 | Jiangxi | JX-2016-120 | mild cases | HFMD |
| 2016 | Sichuan | SC-2016-113 | mild cases | HFMD |
| 2017 | Jiangxi | JX-2017-50 | mild cases | HFMD |
| 2017 | Jiangxi | JX-2017-87 | mild cases | HFMD |
| 2017 | Henan | HeN-2017-130 | mild cases | HFMD |
| 2017 | Hebei | HeB-2017-211 | mild cases | HFMD |
| 2017 | Hebei | HeB-2017-231 | **severe case** | HFMD |

Note: severe cases were marked in bold.
